# Shortcomings in the training program of medical residency during the COVID-19 pandemic in Brazil. How will they be repaired?

**DOI:** 10.1055/s-0041-1728694

**Published:** 2021-04-15

**Authors:** Marcos Felipe Silva de Sá

**Affiliations:** 1 Faculdade de Medicina de Ribeirão Preto, Universidade de São Paulo, Ribeirão Preto, SP, Brazil

Medical Residency Programs (MRPs) have been presented as the dominant model for postgraduate medical training worldwide. They are considered the gold standard by the medical community and give different status to doctors who hold these titles. The success of this model resides in the link established between theoretical learning and the practice experienced in health services, which has made this pattern of specialization an almost mandatory target for the vast majority of medical students, not only for their specialization, but also as a way of correcting eventual deficiencies in undergraduate training. Thus, professional competence in the practice of medicine has become synonymous with specialization that can only be obtained in well-structured programs with a balanced planning between theoretical and practical activities taught by qualified professionals dedicated to these activities.

The COVID-19 pandemic has caused major losses for MRPs. In order to adapt to the negative impacts of the pandemic, MRPs were compulsorily led to a significant reorganization in their schedules. These changes included reductions or cancellations of activities in the operating room and outpatient clinics, visits to wards, simulation sessions, among others. In addition, hospitals have undergone adaptations in their care routines that directly impacted the training of residents in both clinical and surgical areas, since a large part of the program activities were transferred to the care of COVID-19 patients.

On May 8th 2020, the National Medical Residency Commission (Portuguese acronym: CNRM), linked to the Ministry of Education, responsible for the evaluation and accreditation of MRPs throughout Brazil, released a draft Technical Note of recommendations to MRPs for the development of activities during the pandemic. According to the document, each MRP should make its pedagogical project more flexible in order to adapt to the current health reality. Face-to-face classes should be suspended and theoretical activities should be developed on technology-mediated virtual platforms. Video classes and applications that allow interactivity through messages or oral communication were suggested. Regarding the maintenance or suspension of the activities of the medical resident, each MRP should analyze it in a particular way, justifying the decision to the local Medical Residency Commission (Portuguese acronym: Coreme). After the resumption of normality, the replacement of MRP activities not developed during the pandemic would be the object of analysis and subsequent decision by the National Medical Residency Commission. 1

Guidelines were generic and many decisions were delegated to program coordinators themselves, as long as the workload foreseen for residents was respected. However, the pandemic fell like an avalanche over hospital institutions and there was no uniformity of actions to meet the National Medical Residency Commission guidelines. Most services were unprepared for distance learning activities, and the lack of adequate infrastructure for the transmission of video classes, teleconferences and patient care (telemedicine) was an important factor for non-compliance with some of these recommendations.

Several times, this deficiency was solved in an improvised way with many difficulties. Despite these facts, there was progress in many services that considerably improved the teaching system, installed new equipment and invested in the training of teachers/preceptors and coordinators. They also adapted to patient care with the use of telemedicine, reserving face-to-face appointments to cases considered essential due to the severity of the morbidity and/or clinical conditions. 2 Appointments were not scheduled for patients with low-severity morbidities or they were seen via telemedicine consultations.

However, as a result of the worsening of the pandemic across the country, hospitals and outpatient services faced an alarming increase in COVID-19 cases. Frequently clinical directors of hospitals demanded from their local Medical Residency Commission that doctors attending their programs were allocated to supply the human resources shortage to face the pandemic. In addition, in order to protect health professionals, part of the hospital team that presented risk factors was removed from face-to-face activities, generating an important shortage in the teaching and support teams for residents. Therefore, the adaptation process of hospitals to the new situation became quite complicated, which reflected negatively in MRPs and made it impossible to comply with the specific programming for each area.

Considering that surgery is a high-risk situation for the transmission of respiratory infections and following global guidance, both the National Health Agency (ANS) and the National Health Surveillance Agency (Anvisa) advised the postponement of elective and non-essential surgeries, having a considerable impact on the number of surgical procedures. Hundreds of thousands of surgeries were postponed or canceled as a result of this pandemic, causing delay in the diagnosis and treatment of thousands of surgical/oncological cases in this period. 3-6

Despite the negative impact on the training of residents, by mid-2020, a reduction in the course of the pandemic was expected, with progressive return of clinical and surgical activities to normal in the second half of that year. In July 2020, the National Medical Residency Commission made a consultation through a specific questionnaire applied to more than 10,000 medical residents from all Brazilian states and regions. For 73.9% of respondents, it would be possible to regain skills during the period planned for the residency. A similar result was found in the perception of 278 consultants from Medical Residency Commissions. Thus, based on results of the survey, the National Medical Residency Commission decided to maintain the regular start and end dates of Medical Residency Programs for the year 2021, as well as dates foreseen for the selection processes. Exceptional cases related to Medical Residency Programs should be evaluated by the respective supervisors and if an extension of residents' training was necessary, the justification should be forwarded to the National Medical Residency Commission plenary for analysis after approval by the local Medical Residency Commission and the respective State Medical Residency Commission - Cerem) and the guarantee of the scholarship payment by the program itself. Furthermore, even in this condition, the dates of selection processes for the entry of new residents in 2021 would be maintained. 7 The idea with this resolution was the possibility of recovering the competence training during the remaining period planned for the residency. In fact, the programs officially ended on March 1st, 2021 and new classes were admitted to start on that same date. 8

As much as services have endeavored to mitigate the negative impact of the pandemic on Medical Residency Programs, practical activities have been far from the qualitatively and quantitatively ideal. Given this situation, many doubts remain about the final outcome of the training of these residents.

What can be done to recover the training losses of residents enrolled in Medical Residency Programs during the pandemic? Are they going to be put on the job market after finishing a Medical Residency Program with incomplete training? Will they be properly prepared for the exercise of specialties? Will they be entitled to the specialist title awarded at the end of the Medical Residency Program?

Apparently, there are no answers to these questions and, although so many doubts and uncertainties exist about the future of these residents, the topic has not been debated in depth, as it should.

What are the perspectives for residents to complement their specialized training today? There is also no answer to this question neither prospects for a solution to this dramatic situation formed around a generation that had the misfortune to attend the Medical Residency Program in the period of the worst pandemic that has plagued humanity in the last 100 years.

In view of the current situation of the pandemic in Brazil, the horizon for the training of current residents is absolutely bleak in most specialties. The correction of the route will be difficult to execute for economic reasons and the impossibility to postpone the entry of new residents this year, a decision that was taken prematurely already in the middle of 2020.

But, what about 2022? It is time to start preparing for next year and find a way to minimize all the damage done to the training of residents who attended 2020 and 2021 programs. Who should lead this debate? Undoubtedly, the National Medical Residency Commission, as the controller and responsible for the accreditation of programs, should initiate this debate. It must involve representatives of Universities Hospitals which account for the vast majority of programs, the Brazilian Medical Association (Portuguese acronym: AMB) which is official institutions that regulate the activities of specialty societies and the Federal Council of Medicine (Portuguese acronym: CFM), that regulate the activities related to the professional practice of medicine. These last two entities already have a permanent seat on the National Medical Residency Commission.

A study group formed by these four instances on an egalitarian basis would be ideal. This is the most opportune time for any deliberations on the part of the CNRM since we are still in the first quarter of the year 2021 and there would be time for rearrangements in the calendar, particularly with regard to the termination of the current programs, as well as for the entry of new ones classes in 2022.

Obviously, this is a difficult task that no one country in the world has ever experienced it. Therefore, the exchange of information between similar institutions from different countries may be crucial to generate a solution to this impasse. Probably many of these countries are presently discussing and preparing measures to mitigate this problem in the coming months or years and they could share all the knowledge learned during the pandemic.

If none of this happens in Brazil, it may signs that this country does not seem to be caring about the quality of the medical professionals placed in the job market to develop such a noble and relevant mission and whose performance should have a direct impact on the preservation of health and treatment of diseases. In other words, on people's own lives.
